# Sleep and Memory Consolidation Dysfunction in Psychiatric Disorders: Evidence for the Involvement of Extracellular Matrix Molecules

**DOI:** 10.3389/fnins.2021.646678

**Published:** 2021-05-14

**Authors:** Barbara Gisabella, Jobin Babu, Jake Valeri, Lindsay Rexrode, Harry Pantazopoulos

**Affiliations:** Department of Neurobiology and Anatomical Sciences, University of Mississippi Medical Center, Jackson, MS, United States

**Keywords:** extracellular matrix, memory consolidation, schizophrenia, bipolar disorder, PTSD, aging, Alzheimer’s disease, addiction

## Abstract

Sleep disturbances and memory dysfunction are key characteristics across psychiatric disorders. Recent advances have revealed insight into the role of sleep in memory consolidation, pointing to key overlap between memory consolidation processes and structural and molecular abnormalities in psychiatric disorders. Ongoing research regarding the molecular mechanisms involved in memory consolidation has the potential to identify therapeutic targets for memory dysfunction in psychiatric disorders and aging. Recent evidence from our group and others points to extracellular matrix molecules, including chondroitin sulfate proteoglycans and their endogenous proteases, as molecules that may underlie synaptic dysfunction in psychiatric disorders and memory consolidation during sleep. These molecules may provide a therapeutic targets for decreasing strength of reward memories in addiction and traumatic memories in PTSD, as well as restoring deficits in memory consolidation in schizophrenia and aging. We review the evidence for sleep and memory consolidation dysfunction in psychiatric disorders and aging in the context of current evidence pointing to the involvement of extracellular matrix molecules in these processes.

## Introduction

Sleep and circadian rhythm disturbances are emerging as shared features across psychiatric disorders and aging, and are strongly associated with memory consolidation and synaptic dysfunction. Recent studies into the structural and molecular processes of memory consolidation during sleep highlight a large degree of overlap with molecular pathways observed in psychiatric disorders, pointing to the potential of these processes as therapeutic targets for memory dysfunction in psychiatric disorders and aging. Extracellular matrix molecules are critically involved in several of the processes implicated in memory consolidation, and are implicated in psychiatric disorders. Recent work from our group and others points to the involvement of the extracellular matrix in synaptic regulation during sleep. We review the current evidence for sleep and memory consolidation dysfunction in psychiatric disorders and aging, in the context of the potential extracellular matrix molecules as a key molecules in memory consolidation dysfunction.

## Sleep and Memory Consolidation

The concept that memory is strengthened during sleep has existed for centuries, at least since early descriptions from the Roman scholar Quintilian, who observed improved memory after sleep (Butler, 1921). The formal concept of memory consolidation was first introduced by Muñller and Pilzecker who introduced the term ‘consolidation’ to describe the process by which memory is strengthened over time, rather than forming as instant permanent memories (Muller and Pilzecker, 1900). In 1924, the seminal work by Jenkins and Dellenbach (1924) was the first formal study demonstrating memory consolidation during sleep, reporting improved declarative memory for nonsense word syllables after sleep. Modern advances in techniques have allowed for a broad range of studies that provide insight into the potential mechanisms behind memory consolidation during sleep. Collectively, these studies have resulted in the emergence of several current, partially complementary theories regarding the process of memory consolidation.

### Current Concepts of Memory Consolidation

A growing collection of studies from Tononi and Cirelli have established the synaptic homeostasis hypothesis of sleep (Tononi and Cirelli, 2006, 2014). This hypothesis proposes that neurons form and strengthen new synapses during wakefulness, as organisms interact with their environment and encode new memories. In this manner, neurons potentiate synapses during wakefulness in response to environmental inputs, and environmental interactions provide feedback regarding the accuracy of these synapses. During sleep, when the active encoding process is offline, synapses are downscaled, in order to enhance the signal to noise ratio, thus improving memory function (Tononi and Cirelli, 2006, 2014). Several studies supporting this concept have reported decreased dendritic spines and synapses during sleep in sensory and motor cortical regions in rodents (Maret et al., 2011; de Vivo et al., 2017), as well as in the drosophila brain (Gilestro et al., 2009; Bushey et al., 2011) and gene expression changes in pathways involved in synaptic downscaling during sleep (Cirelli, 2005). A growing number of recent studies suggest that synaptic downscaling occurs specifically during REM sleep (Li et al., 2017; Zhou et al., 2020). The firing rates of fast-firing inhibitory neurons expressing the calcium binding protein parvalbumin increases during REM sleep (Niethard et al., 2016), possibly contributing to synaptic downscaling by increasing inhibitory tone.

Despite the evidence for synaptic downscaling, an increasing number of studies suggest that sleep is also important for strengthening specific synapses (Vecsey et al., 2009; Yang et al., 2014; Havekes et al., 2016). Evidence that sleep deprivation impairs memory strength (Vecsey et al., 2009), and results in decreased hippocampal dendritic spines (Havekes et al., 2016), supports the hypothesis that selective synapses are strengthened during sleep. Furthermore, sleep deprivation results in decreases of dendritic spines (Raven et al., 2019) as well as the number of granule cells expressing the immediate early gene Arc in the mouse dentate gyrus (Delorme et al., 2019). However, a recent electron microscopy study reporting increased synaptic density and axon-spine interface in the hippocampus of sleep deprived mice (Spano et al., 2019) points to synaptic downscaling in the hippocampus. These discrepancies may be due in part to region and branch specific regulation of dendritic spines during sleep. Our recent study in the CA1 hippocampal area showing branch specific changes in dendritic spines following sleep deprivation (Gisabella et al., 2020) provides support for this branch and region specific regulation. Further support comes from a live imaging study of dendritic spines in the motor cortex. This work demonstrates that sleep promotes dendritic spine formation of motor memory in selective branches of layer V motor cortex neurons (Yang et al., 2014), These studies support the hypothesis that sleep is involved in strengthening selective synapses formed during wakefulness for selective memories, and this occurs even in cortical areas where net synaptic downscaling during sleep has been reported (Maret et al., 2011; de Vivo et al., 2017). Such discrepancies have also been proposed to arise from methodological differences, particularly differences in sleep deprivation methods (Havekes and Aton, 2020). An extensive review of mouse sleep deprivation studies highlights the role of novelty exposure in sleep deprivation methods as a potential major methodological difference contributing to discrepancies in synaptic downscaling or upscaling findings following sleep deprivation (Havekes and Aton, 2020). Differences in circadian time of sampling as well as sex differences are also proposed to contribute to these discrepancies between studies (Havekes and Aton, 2020).

In summary, evidence suggests that while sleep results in a net reduction of synapses in certain brain regions, specific synapses in these regions are selectively potentiated during sleep. Furthermore, this net loss of synapses in sensory and motor cortical areas may not apply to all brain regions. A current theory by Rasch and Born proposes that certain synapses formed during the day are tagged by specific proteins that indicate these synapses will be strengthened during sleep, whereas other synapses are pruned as memory storage is reorganized (Rasch and Born, 2013; Dudai et al., 2015). According to this theory, memories are reorganized during slow wave sleep in a process called systemic consolidation (Rasch and Born, 2013; Dudai et al., 2015). In systemic consolidation, memory representations are reactivated, and transferred from short-term storage sites such as the hippocampus, to long-term storage in neocortical areas and integrated into existing knowledge schemas (Rasch and Born, 2013; Dudai et al., 2015). Memories are then strengthened in these long-term storage areas during REM sleep, in a process called synaptic consolidation, while the short-term storage memories are removed via synaptic pruning (Rasch and Born, 2013; Dudai et al., 2015). An increasing number of studies have shown that neuronal network activation in the hippocampus during spatial learning is replayed in the same sequence during sleep, particularly during slow wave sleep (Pavlides and Winson, 1989; Wilson and McNaughton, 1994; Skaggs and McNaughton, 1996; Kudrimoti et al., 1999; Poe et al., 2000; Bendor and Wilson, 2012). Hippocampus-guided neuronal network replay during sleep has now been reported in several brain regions, including the striatum (Lansink et al., 2009) and the medial prefrontal cortex (Euston et al., 2007). These studies provide support for the theory of systemic consolidation during slow wave sleep. Further support for this hypothesis comes from animal studies showing that newly formed memories become independent of the hippocampus faster and are more stable when associated with pre-existing neocortical schema (Tse et al., 2007; Tse et al., 2011).

Which synapses are strengthened during sleep, and how they are distinguished from synapses that are downscaled? Growing evidence suggests that certain synapses are tagged in an activity dependent manner. During sleep, while encoding is offline, memories tagged during wakefulness can be consolidated through systemic consolidation and synaptic consolidation, whereas non-tagged synapses can be pruned, greatly reducing the signal to noise ratio and restoring the capacity to form new synapses (Benchenane et al., 2010; Inostroza and Born, 2013). The level of coherence in theta frequency in networks involved in formation of specific memories during encoding has been proposed to be associated with tagging selective memories during wakefulness to be consolidated during sleep (Benchenane et al., 2010; Inostroza and Born, 2013). Studies discussed below provide some insight into the potential molecular mechanisms behind these proposed memory consolidation processes.

### Molecular Mechanisms of Memory Consolidation

Long-term plasticity (LTP) has been proposed to be associated with local tagging of synapses (Frey and Morris, 1997). These tags capture synthesized plasticity related proteins at selective dendritic spines, which allow for stabilization of these spines (Redondo and Morris, 2011). CamKII dependent remodeling of the actin cytoskeleton in dendritic spines is an essential feature for synaptic tagging for memory enhancement (Ramachandran and Frey, 2009; Redondo et al., 2010). The proposed molecular mechanisms of synaptic tagging for memory consolidation during sleep may differ from the mechanisms described for memory enhancement during wakefulness. Dopaminergic signaling, implicated in several psychiatric disorders (Laruelle et al., 1999; Abi-Dargham et al., 2000; Berke and Hyman, 2000; Hyman and Malenka, 2001; Volkow et al., 2004; Tremblay et al., 2005; Volkow et al., 2006; Abi-Dargham et al., 2009; Cousins et al., 2009; Anand et al., 2011; Belujon and Grace, 2017; Pizzagalli et al., 2019), may contribute to tagging memories for subsequent consolidation during sleep. Increased activation of dopaminergic reward circuits results in enhanced memory reactivation during slow wave sleep in rodents (Gomperts et al., 2015), and enhanced memory consolidation in humans (Feld et al., 2014).

FOXO6 is a transcription factor expressed in the hippocampus (Jacobs et al., 2003; Hoekman et al., 2006; Salih et al., 2012), as well as the amygdala and cortex (Salih et al., 2012), and is implicated in memory consolidation (Salih et al., 2012). FOXO6 knockout mice show selective impairments in contextual and object recognition memory, together with decreased density of dendritic spines in CA1 of the hippocampus (Salih et al., 2012). Furthermore, these mice display altered hippocampal theta wave frequencies during exploration of novel objects, suggesting decreased synchronization of neurons during exploration (Salih et al., 2012). A deficit in decreased synchronization may indicate decreased synaptic tagging, contributing to subsequent memory consolidation deficits (Benchenane et al., 2010; Inostroza and Born, 2013).

The immediate early gene Arc is another marker located in dendritic spines, particularly in sites of increased postsynaptic activity (Steward et al., 1998). Arc knockout mice have normal short term memory, together with impaired long-term memory consolidation (Plath et al., 2006). Furthermore, amygdala specific knockdown of Arc blocks fear memory consolidation (Ploski et al., 2008). In turn, Arc mutant mice display deficits in LTP and LTD (Nikolaienko et al., 2018). Sleep deprivation results in brain region specific changes in Arc expression in the mouse brain (Delorme et al., 2019), providing further support for a complex role in the regulation of synaptic plasticity during sleep. Recent studies have identified Homer1a as a potential tag for synaptic pruning during sleep (Diering et al., 2017). Homer1a signaling through metabotropic glutamate receptors was shown to be necessary for synaptic downscaling during sleep (Diering et al., 2017). Furthermore, sleep deprivation has been shown recently to cause increased phagocytosis of dendritic spines by astrocytes, together with increased activation of microglia (Bellesi et al., 2017). This enhanced spine elimination during sleep deprivation may reflect mechanistic processes that are also involved in synaptic downscaling during sleep at a less pronounced level.

### Extracellular Matrix: Evidence for Involvement in Memory Consolidation

**FIGURE 1:**
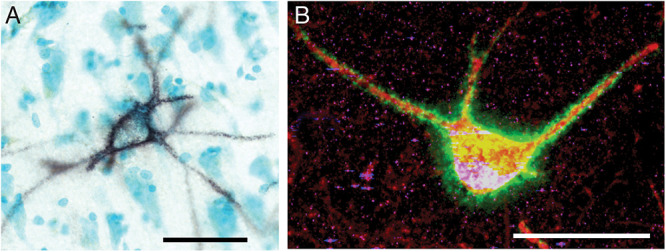
Perineuronal nets in the human and mouse hippocampus. **(A)** Photomicrograph of a representative perineuronal net labeled by wisteria floribunda agglutinin in the human hippocampus. Cells are counterstained with methyl green according to previously published methods (Pantazopoulos et al., 2010b). **(B)** Confocal image of a perineuronal net in the mouse hippocampus labeled with wisteria floribunda agglutinin (green), surrounding a parvalbumin positive neuron (red) co-expressing synapsin-1 (blue). Scale bars = 50 μm.

Extracellular matrix molecules (ECM) interact with many of the synaptic regulation processes described above, and a growing amount of evidence points to the involvement of ECMs in memory consolidation (Table 1). Chondroitin sulfate proteoglycans (CSPGs) are a family of ECMs that are critically involved in a range of neurodevelopmental and adult functions (Dobbertin et al., 2003; Garwood et al., 2003; Wu et al., 2004; Maeda et al., 2010; Maeda et al., 2011). In late postnatal development, these molecules contribute to the formation of perineuronal nets (PNNs) (Figure 1), ECM structures that surround subpopulations of neurons, first described by Camillo Golgi in 1898 (Golgi, 1989). Work over the past several decades has elucidated the critical role of these structures in a broad range of processes, including synaptic plasticity and regulation of electrophysiological properties (Kalb and Hockfield, 1988; Sugiyama et al., 2008; Gogolla et al., 2009). PNNs are involved in regulating several functional neuronal properties, including somatic inhibition, glutamate receptor trafficking, and synaptic stabilization (Pizzorusso et al., 2002; Frischknecht et al., 2009; Frischknecht and Gundelfinger, 2012; Carceller et al., 2020; Christensen et al., 2021). For example, removal of PNNs decreases densities of inhibitory and excitatory synapses on the soma of PVB neurons (Carceller et al., 2020), reduces gamma activity (Christensen et al., 2021), and de-stabilizes the grid cell network (Christensen et al., 2021). Several studies, including seminal work in the visual cortex and the amygdala, demonstrated that PNNs form during the end of critical periods of plasticity and mark the closure of these periods by conferring an adult form of restricted plasticity (Pizzorusso et al., 2002; Gogolla et al., 2009).

**TABLE 1:** Evidence for ECM molecules in sleep and memory consolidation processes.

**Reference**	**Summary of Findings**
Taishi et al., 2001	Sleep deprivation results in decreased mRNA expression and enzymatic activity of MMP-9
Szklarczyk et al., 2002	Dendritic spine remodeling coincides with increased MMP-9 expression
Nagy et al., 2006	MMP-9 expression is crucial in late-stage LTP and hippocampal-dependent memory
Nagy et al., 2007	Disrupted MMP-3 and MMP-9 activity in the hippocampus causes avoidance learning impairments
Brown et al., 2009	Pharmacological disruption of MMP activity results in disruption of fear memory reconsolidation
Beurdeley et al., 2012	Inhibition of OTX2 and PNN binding enhances synaptic plasticity and reduces PNN and PVB expression
Hayashi et al., 2013a	Cathepsin-S KO mice lack diurnal rhythms in dendritic spine density
Niethard et al., 2016	Increased PVB neuron firing rates are accompanied by decreased pyramidal neuron activity during REM sleep
Harkness et al., 2019	PNN composition is increased in the prelimbic cortex of sleep-deprived rats, suggesting that PNN composition decreases during sleep
Shi et al., 2019	PNN degradation contributes to GABAergic inhibition on hippocampal pyramidal neurons, contributing to LTP and memory consolidation impairment
Delorme J. et al., 2020	Gene expression for CSPG synthesis and degradation pathways is altered in mice sleep deprived following fear conditioning
Harkness et al., 2020	Diurnal rhythms of PNN intensity and OTX2 expression occurs in the rat medial prefrontal cortex, with decreased PNN intensity and OTX2 expression during sleep.
Nguyen et al., 2020	IL-33 signaling stimulates ECM engulfment by microglia, promoting experience-dependent synaptic plasticity
Pantazopoulos et al., 2020a	PNN composition decreases during sleep and increases during wakefulness; sleep deprivation prevents decreases in PNN composition
Pantazopoulos et al., 2020a	The CSPG protease cathepsin-S is expressed in a diurnal manner by microglia in the mouse brain, antiphase to PNN composition rhythms.

Perineuronal nets have been historically considered as very stable structures, and the holes in PNNs are hypothesized as storage sites for long term memories (Tsien, 2013). However, if PNNs are such highly stable structures that restrict plasticity, how are new memories formed, and how are they strengthened during sleep? PNNs would need to be modified to weaken their restrictive properties during sleep in order for synaptic downscaling and/or synaptic reorganization to occur for memory consolidation. Several studies suggest that endogenous proteases modify PNNs during learning to allow for formation of synapses (Nagy et al., 2007; Brown et al., 2009; Ganguly et al., 2013; Banerjee et al., 2017; Slaker et al., 2018). A number of endogenous proteases expressed in the adult brain can cleave chondroitin sulfate proteoglycans (CSPGs), key components of PNNs. Endogenous proteolytic remodeling of ECM components, including CSPGs, is mediated by several proteases including matrix metalloproteases (MMPs), ‘a disintegrin and metalloproteases’ (ADAMs) and ‘a disintegrin and metalloproteases with a thrombospondin motif (ADAMTS) (Muir et al., 2002; Medina-Flores et al., 2004; Hobohm et al., 2005; Porter et al., 2005; Bajor and Kaczmarek, 2013). Several of these proteases, such as MMP-9, modify ECM composition during learning tasks, possibly allowing for formation of new synapses in response to environmental stimuli (Nagy et al., 2007; Brown et al., 2009; Ganguly et al., 2013). For example, MMP-9 expression is induced in the amygdala, prefrontal cortex, and hippocampus during contextual fear learning (Ganguly et al., 2013). Pharmacological disruption of MMP activity results in disruption of reconsolidation of fear memory, suggesting that MMP expression is a necessary component for this process (Brown et al., 2009). Similar disruption of MMP-3 and MMP-9 activity in the hippocampus results in impairment of spatial memory (Wright et al., 2007) and avoidance learning (Nagy et al., 2007).

These effects may be through regulation of synaptic stabilization. For example, increased MMP9 expression has been shown to coincide with dendritic spine remodeling (Szklarczyk et al., 2002), and MMP9 is required for late-stage LTP and hippocampal dependent memory (Nagy et al., 2006). *In vivo* and *in vitro* studies suggest that PNNs stabilize synaptic strength. An increasing number of studies suggest that CSPGs and PNNs are critically involved in the regulation of synaptic plasticity. For example, electrophysiological recordings from *in vitro* mouse hippocampal slices treated with chondroitinase ABC to remove CSPGs, show a two-fold decrease in long-term potentiation (LTP) but not in short-term plasticity (Bukalo et al., 2001). A similar decrease of LTP was observed in mice lacking a key PNN component, tenascin-R, suggesting that CSPG regulation of long-term synaptic plasticity occurs through modulation of PNN composition (Bukalo et al., 2001). The strength of hippocampal LTP has been shown to vary in a circadian manner (Chaudhury et al., 2005), and PNNs regulate synaptic strength and stability (Bukalo et al., 2001; Brakebusch et al., 2002; Geissler et al., 2013) suggesting that PNN composition may be regulated in a circadian manner, potentially through proteases.

**FIGURE 2:**
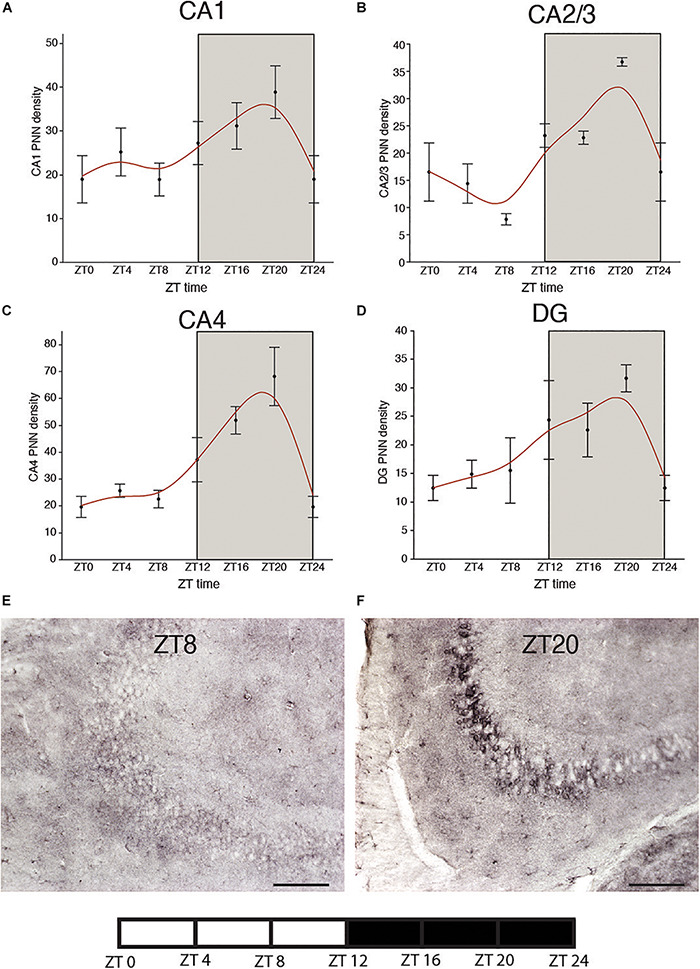
Diurnal rhythms of chondroitin sulfate synthase 1 labeled perineuronal nets in the mouse hippocampus. CHSY1 immunoreactive PNNs were labeled with rabbit anti-CHSY1antibody (cat#14420-1-AP, ProteinTech) at 1:500 μl dilution. Immunolabeling and quantification was conducted on serial sections from the same mice in our previous study according to the same methods described in detail (Pantazopoulos et al., 2020a). Diurnal rhythms of chondroitin sulfate synthase 1 (CHSY1) immunoreactive PNNs were observed in hippocampal sector CA1 **(A)** CA2/3 **(B)**, CA4 **(C)** and the dentate gyrus **(D)**, with peaks at ∼ZT20 and troughs at ∼ZT8. Error bars represent standard deviation. Representative low magnification images of CHSY1 labeling in the mouse hippocampus at ZT 8 **(E)** and ZT 20 **(F)**.

**FIGURE 3:**
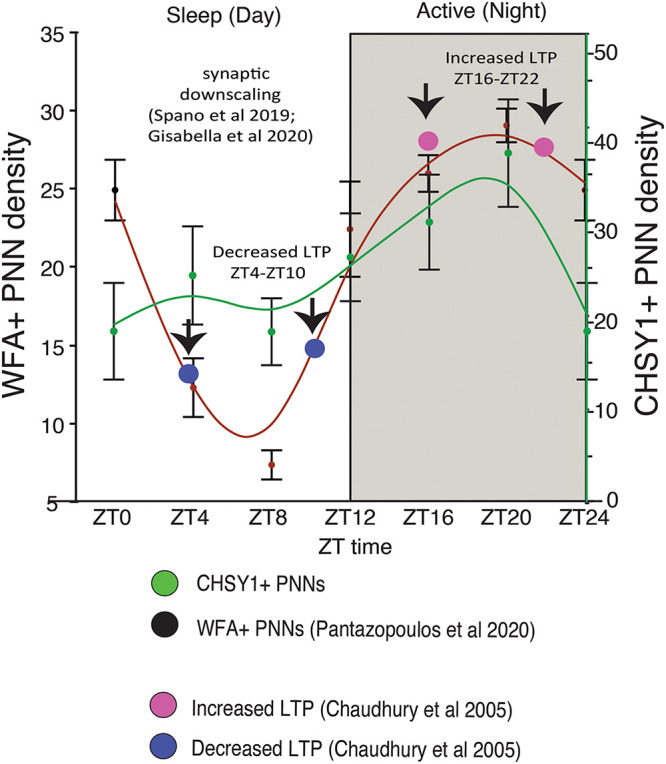
Diurnal PNN Rhythms Coincide with Rhythms in CHSY1 Expression and Memory Consolidation Processes. Diurnal rhythms in the numerical density of WFA+ PNNs decreases during the day as mice sleep, reaching the lowest density in WFA+ PNN numbers between ZT 4-ZT 10 (red curved line). This coincides with decreased expression of CHSY1 PNNs (green curved line) and the reported daytime decrease in LTP [blue circles, (Chaudhury et al., 2005)]. This decrease in PNNs also coincides with reported broad synaptic downscaling in this region (Spano et al., 2019; Gisabella et al., 2020). In comparison, the numerical density of WFA+ and CHSY1 immunoreactive PNNs peaks during the dark at ∼ZT 20 during the active period for nocturnal mice, coinciding with the reported increase in LTP at night in mice [pink circles, (Chaudhury et al., 2005)]. These results suggest that decreases in PNN composition during sleep coinciding with synaptic downscaling and decreased LTP, potentially allowing for memory consolidation. PNN composition is restored during the active wake periods, in part through CHSY1 expression.

We recently reported circadian rhythms in PNN composition in the mouse brain, with lower densities of wisteria floribunda agglutinin (WFA) labeled PNNs during sleep and higher densities during wakefulness (Pantazopoulos et al., 2020a). PNNs labeled by chondroitin sulfate synthase 1 antibodies display rhythms in densities of CHSY-immunoreactive PNNs in the hippocampus that mirror WFA labeled PNN densities in the same animals (Figures 2, 3), suggesting that some components of PNNs are produced in a circadian manner. Furthermore, 5 h of sleep deprivation prevented decreases in PNN densities (Pantazopoulos et al., 2020a). Recent work shows similar increases in PNN composition in the prelimbic cortex of sleep deprived rats and provides further support that PNN composition is decreased during sleep (Harkness et al., 2019). Our PNN labeling and quantification method represents the number of WFA labeled PNNs above a certain detection threshold as weakly labeled PNNs do not meet detection criteria. We interpret our results as altered PNN composition rather than fluctuating numbers of PNNs. Similar diurnal rhythms in PNN composition were reported recently in the rat medial prefrontal cortex (Harkness et al., 2020). Decreased PNN composition during sleep (Pantazopoulos et al., 2020a) coincides with reduced LTP amplitude during the light phase in the hippocampus (Chaudhury et al., 2005). Further evidence for the involvement of ECM molecules including CSPGs in memory consolidation during sleep comes from a recent single cell gene expression profiling study describing altered gene expression of CSPG synthesis and degradation pathways in CamKIIα neurons when animals that slept normally were compared to sleep deprived animals following fear conditioning (Delorme J. et al., 2020).

**FIGURE 4:**
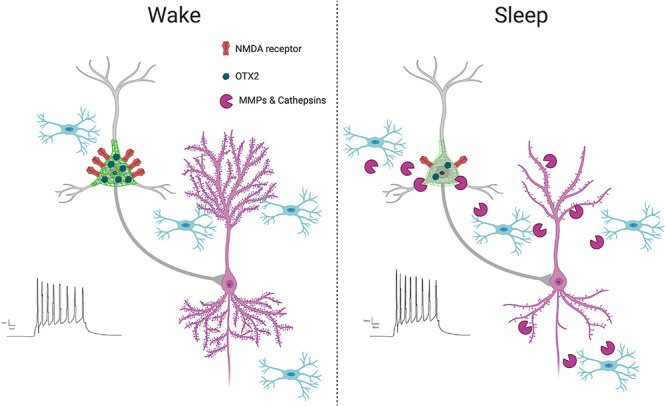
PNN intensity during wakefulness vs. sleep. PNNs display increased intensity during wakefulness (Harkness et al., 2020; Pantazopoulos et al., 2020a), coinciding with increased glutamatergic receptor activity (Harkness et al., 2020) and OTX2 expression (Harkness et al., 2020). This condition promotes LTP as new synapses are formed when interacting with the environment in active states, reflected by increased LTP (Chaudhury et al., 2005) and dendritic spine numbers on pyramidal neurons (Spano et al., 2019; Gisabella et al., 2020). In contrast, PNN intensity decreases during sleep, coinciding with several factors involved in synaptic regulation, including increased expression of proteases in part by microglia (Taishi et al., 2001; Pantazopoulos et al., 2020a), decreased OTX2 protein and glutamatergic receptor activity (Harkness et al., 2020), increased PVB neuron firing (Niethard et al., 2016), decreased LTP (Chaudhury et al., 2005) and decreased dendritic spines on pyramidal neurons, as memories are consolidated during synaptic downscaling (Spano et al., 2019; Gisabella et al., 2020).

Perineuronal nets may contribute to synaptic regulation in several aspects (Figure 4). For example, PNNs may be involved in circadian regulation of synaptic strength through their regulation of glutamatergic receptors. ECMs regulate glutamate receptor trafficking (Frischknecht et al., 2009; Frischknecht and Gundelfinger, 2012). Long-term (72 h) of sleep deprivation has been shown to result in decreased long-term potentiation (LTP), a form of synaptic plasticity that is associated with memory consolidation (Martin et al., 2000) in CA1 hippocampal neurons. Furthermore, short term (5 h) of sleep deprivation has also been shown to disrupt LTP (Vecsey et al., 2009; Havekes et al., 2016). Reduction in LTP was associated with reduced NMDA receptor activity and surface expression of the NR1 and NR2A subunits of the NMDA receptor in sleep deprived rats, indicating that sleep deprivation prevents translocation of NMDA receptors to the cell surface (McDermott et al., 2006). Reduction in LTP associated with trafficking of NMDA receptors to the surface of CA1 neurons has also been shown following 24 h of sleep deprivation (Chen et al., 2006). Day/night differences in the AMPA/NMDA ratio were reported recently in the rat medial prefrontal cortex, with increased AMPA/NMDA ratio during the night (ZT18) compared to the day (ZT6). This was accompanied by increased vGlut1 glutamatergic synapses on PVB neurons at ZT18 and increased PNN intensity (Harkness et al., 2020).

Circadian rhythms of PNN composition (Harkness et al., 2020; Pantazopoulos et al., 2020a) may also contribute to memory consolidation through the regulation of PVB neuron firing properties. GABAergic inhibition of excitatory neurons is proposed to contribute to synaptic downscaling by promoting long term depression (LTD) (Niethard et al., 2016), in agreement with reported decreased LTP amplitude during the light phase (Chaudhury et al., 2005). PVB neuron firing rates increase during sleep, particularly during REM sleep, accompanied by overall reduction in pyramidal neuron activity (Niethard et al., 2016). Removal of PNNs through chondroitinase ABC treatment has been shown to enhance frequency of spontaneous inhibitory postsynaptic currents on hippocampal pyramidal neurons, which contributes to impairment of LTP and fear memory consolidation (Shi et al., 2019), suggesting PNN degradation enhanced PVB neuron inhibition of pyramidal cells. However, PVB neuron activity is also essential for memory consolidation (Ognjanovski et al., 2017; Xia et al., 2017), suggesting a more complex role of PNN regulation of PVB neuron activity in physiological conditions, possibly reflected by more subtle changes in PNN composition by endogenous proteases rather than broad PNN degradation caused by chABC administration. Diurnal and circadian fluctuations in excitatory/inhibitory balance have also been reported in rodents and humans (Chellappa et al., 2016; Bridi et al., 2020). Circadian rhythms in PNN composition may contribute to daily rhythms in excitatory/inhibitory balance through regulation of PVB neuron firing as well as regulation of excitatory and inhibitory synapses on PVB soma (Carceller et al., 2020; Harkness et al., 2020). Taken together, these data suggest that PNN composition changes during sleep may in part contribute to synaptic downscaling by allowing increased inhibitory inhibition for synapse de-potentiation by PVB neurons.

Several factors may contribute to circadian regulation of PNN composition (Figure 4). Sleep deprivation has been shown to decrease both mRNA expression and enzymatic activity of MMP9 (Taishi et al., 2001), suggesting that increased MMP-9 activity during sleep may contribute to PNN composition changes. MMPs, including MMP-9, are primarily produced by astrocytes and microglia in the brain (Rosenberg et al., 2001; Cahoy et al., 2008). Recent studies point to the involvement of microglia in degrading ECM components during sleep. A large number of recent studies have established the role of microglia in the regulation of synaptic plasticity in the adult brain (Tremblay et al., 2010; Paolicelli et al., 2011; Schafer et al., 2012, 2014; Miyamoto et al., 2013; Wake et al., 2013; Kim et al., 2017; Bolós et al., 2018; Stevens and Schafer, 2018). For example, microglia continuously sense synapses in areas of high synaptic activity, monitoring the synaptic environment and prune synapses (Tremblay et al., 2010; Paolicelli et al., 2011; Schafer et al., 2012, 2014; Miyamoto et al., 2013; Wake et al., 2013; Kim et al., 2017; Stevens and Schafer, 2018). Microglial dysfunction in the adult hippocampus has been shown to result in a reduction of dendritic spines along with increased ECM expression (Bolós et al., 2018), suggesting microglia participate in degrading the ECM to allow for increased synaptic plasticity. This process may be under control of the molecular circadian clock, as suggested by studies describing that the circadian molecular clock in microglia regulates several processes including microglial morphology, immune response, and synaptic processing (Hayashi et al., 2013a, b; Fonken et al., 2015).

Cathepsin-S may represent one of the components through which microglia regulate PNN composition in a circadian manner. Cathepsin-S is a protease produced by microglia reported to cleave several CSPGs (Petanceska et al., 1996; Pantazopoulos et al., 2020a). Cathepsin-S knockout mice, in which diurnal rhythms of dendritic spine density were reported (Hayashi et al., 2013a), also display deficits in novel object recognition (Takayama et al., 2017), supporting the hypothesis that cathepsin-S rhythms regulate key roles of PNNs in memory processing. Our evidence for circadian rhythms of cathepsin-S expression in microglia in the mouse brain, antiphase to PNN rhythms, and evidence that cathepsin-S reduces PNN labeling, suggests that increased expression of cathepsin-S proteases from microglia during sleep are involved in weakening PNN composition (Pantazopoulos et al., 2020a), potentially allowing for synaptic remodeling during memory consolidation. Recent evidence that microglia promote synaptic plasticity by degrading ECM molecules including the key PNN components aggrecan and brevican through cathepsin-C and other proteases (Nguyen et al., 2020) provides further support that microglia are involved in mediating changes in PNN composition during sleep to allow for memory consolidation processes.

Several studies point to multiple pathways in which neurons and microglia may modify PNN composition (Bertocchi et al., 2020; Harkness et al., 2020; Nguyen et al., 2020; Pantazopoulos et al., 2020a). Neurons signal to microglia through IL-33 to promote experience dependent synaptic plasticity in part by stimulating engulfment of ECMs (Nguyen et al., 2020). This process is impaired in IL-33 deficient mice, resulting in impaired spatial memory precision (Nguyen et al., 2020). Neuropeptide-Y (NPY) signaling is also involved in regulating hippocampal PNN composition to allow for plasticity during spatial learning (Bertocchi et al., 2020). Conditional loss of NPY receptors leads to increased PNN intensity in the hippocampus, associated with impaired spatial learning, and enzymatic digestion of PNNs restores learning abilities (Bertocchi et al., 2020). In the rat medial prefrontal cortex, increases in PNN intensity during the night were accompanied by increased OTX2 expression in PVB cells with and without PNNs (Harkness et al., 2020). OTX2 has been shown to target PVB cells through binding of a 15 amino acid peptide sequence within OTX2 to di-sulfated chondroitin sulfate (CS-D and CS-E) in PNNs (Beurdeley et al., 2012). Blocking this peptide sequence reduces PNN and PVB expression and enhances synaptic plasticity (Beurdeley et al., 2012), suggesting that OTX2 may contribute to the circadian regulation of PNN composition. Furthermore, studies in xenopus suggest that OTX2 influences CLOCK expression in a positive, bidirectional manner (Green et al., 2001; Morgan, 2002). Together these studies suggest OTX2-CLOCK interactions may contribute to circadian PNN composition rhythms and in turn synaptic reorganization during sleep.

In addition to PNNs, CSPGs are also located on perisynaptic regions and several ECM structures that may regulate synaptic plasticity. For example, perisynaptic aggregates of the CSPGs aggrecan and brevican have been reported around neurons devoid of PNNs (Lendvai et al., 2012, 2013). Brevican is enriched in synaptic membrane protein fractions (Seidenbecher et al., 1995) and is essential for hippocampal synapse maturation (Favuzzi et al., 2017). Brevican has been shown to be critically involved in the regulation of synaptic plasticity (Yuan et al., 2002; Blosa et al., 2015; Sonntag et al., 2018; Gottschling et al., 2019; Mitlohner et al., 2020) and cleavage of BCAN by ADAMTS4 increases in the rodent hippocampus following excitotoxic lesion, coinciding with reduced synaptic density (Yuan et al., 2002). CSPG structures formed by clusters of chondroin-6 sulfate have also been described in several species (Hayashi et al., 2007; Horii-Hayashi et al., 2010; Pantazopoulos et al., 2015), and are proposed to contribute to the regulation of synaptic plasticity (Hayashi et al., 2007; Horii-Hayashi et al., 2010; Chelini et al., 2018). Alterations in circadian rhythms of endogenous ECM proteases and ECM molecules thus may impact memory consolidation processes in several manners, including through ECM regulation of dendritic spines in excitatory neurons.

## Memory Consolidation and Extracellular Matrix Molecules in Psychiatric Disorders

Disturbances in sleep and circadian rhythms, along with memory dysfunction are two key features shared across many psychiatric disorders, including schizophrenia (SZ), major depression disorder (MDD), bipolar disorder (BD), post-traumatic stress disorder (PTSD) and Alzheimer’s Disease (Bliwise, 1993; Hofman et al., 1996; Leibenluft et al., 1996; Colombo et al., 1999; Lin et al., 2000; Van Cauter et al., 2000; Huang et al., 2002; Mellman et al., 2002; Jackson et al., 2003; Harvey et al., 2005; Armitage, 2007; Benedetti et al., 2007; Ferrarelli et al., 2007; Wang and Teichtahl, 2007; Manoach et al., 2010, 2014, 2016; McCarthy et al., 2012, 2013, 2016, 2019; Bunney and Bunney, 2013; Burton et al., 2013; Li et al., 2013; McClung, 2013; Palagini et al., 2013; Parsons and Ressler, 2013; Colrain et al., 2014; Medina et al., 2014; Narishige et al., 2014; Bunney et al., 2015; Wang et al., 2015; Chen et al., 2016; Pagani et al., 2016; Pantazopoulos et al., 2017; Nudell et al., 2019; Koob and Colrain, 2020). Furthermore, alterations in ECMs (Table 2) and synaptic markers have also been reported across these disorders (DeKosky and Scheff, 1990; Terry et al., 1991; Scheff and Price, 1993; Woo et al., 2004; Dorph-Petersen et al., 2009; Feyissa et al., 2009; Sweet et al., 2009; Kristiansen et al., 2010; Schmitt et al., 2010; Vrajova et al., 2010; Penzes et al., 2011; Duman and Aghajanian, 2012; Kang et al., 2012; Duric et al., 2013; Glausier and Lewis, 2013; Licznerski and Duman, 2013; Konopaske et al., 2014, 2015; Shelton et al., 2015; MacDonald et al., 2017).

**TABLE 2:** Evidence for ECM molecules and PNNs in psychiatric disorders.

**Reference**	**Psychiatric Disorder of Interest**	**Summary of Findings**
Morawski et al., 2004; Morawski et al., 2010	Alzheimer’s Disease (AD)	PNNs protect neurons in Alzheimer’s Disease patients
Baig et al., 2005	Alzheimer’s Disease (AD)	WFA labeled perineuronal nets are decreased in the frontal cortex of patients with AD
Nagy et al., 2006	Post-Traumatic Stress Disorder (PTSD)	MMP9 KO mice display fear memory and LTP deficits
Wright et al., 2007	Substance Use Disorders (SUD)	Ethanol impairs spatial memory and reduces MMP-9 levels.
Brown et al., 2007	Substance Use Disorders (SUD)	Broad-spectrum MMP inhibition disrupts both acquisition and consolidation of cocaine reward memory in rodents
Mizoguchi et al., 2007	Substance Use Disorders (SUD)	MMP-9 inhibition reduces methamphetamine induced conditioned place preference and nucleus accumbens dopamine levels
Brown et al., 2009	Post-Traumatic Stress Disorder (PTSD)	Intracerebroventricular administration of the MMP inhibitor FN-439 disrupts fear memory reconsolidation in rodents
Samochowiec et al., 2010	Substance Use Disorders (SUD)	MMP-9 polymorphism is associated with alcoholism
Pantazopoulos et al., 2010b	Bipolar Disorder (BD) and Schizophrenia (SZ)	WFA labeled PNNs are decreased in the amygdala and entorhinal cortex of subjects with SZ
Mauney et al., 2013	Bipolar Disorder (BD) and Schizophrenia (SZ)	WFA labeled PNNs are decreased in the prefrontal cortex of subjects with SZ
Pantazopoulos et al., 2014	Bipolar Disorder (BD) and Schizophrenia (SZ)	WFA labeled PNNs are decreased in the hippocampus of subjects with SZ
Pantazopoulos et al., 2015	Bipolar Disorder (BD) and Schizophrenia (SZ)	Aggrecan labeled PNNs are decreased in the amygdala of subjects with SZ, and CS-6 labeled PNNs are decreased in the amygdala of subjects with SZ and BD
Enwright et al., 2016	Bipolar Disorder (BD) and Schizophrenia (SZ)	Intensity of WFA labeling is reduced in the dorsolateral prefrontal cortex of subjects with SZ
Steullet et al., 2017	Bipolar Disorder (BD) and Schizophrenia (SZ)	WFA labeled PNNs are reduced in the thalamic reticular nucleus in subjects with SZ and in subjects with BD
Smith et al., 2011	Substance Use Disorders (SUD)	MMP inhibition reduced escalated ethanol self-administration in rodents
Shah and Lodge, 2013	Substance Use Disorders (SUD)	PNN degradation in the ventral hippocampus enhances dopamine VTA activity and amphetamine response
Kovatsi et al., 2013	Substance Use Disorders (SUD)	Subjects with chronic heroin use have altered blood serum levels of MMP-9, MMP-2, TIMP-1 and TIMP-2
Natarajan et al., 2013	Substance Use Disorders (SUD)	Nicotine induces transient changes in MMP-2, 3 and 9 in the rodent hippocampus, and MMP inhibition impairs nicotine conditioned place preference
Guirado et al., 2014	Major Depression Disorder (MDD)	PNN reduction and dendritic spine and synaptic markers alterations occur in mice subjected to chronic fluoxetine treatment; fluoxetine efficacy may occur through PNN degradation to promote synaptic plasticity
Smith et al., 2014	Substance Use Disorders (SUD)	MMP activity is required for cocaine relapse in rodents
Slaker et al., 2015	Substance Use Disorders (SUD)	PNNs in the rodent prefrontal cortex are required for acquisition and reconsolidation of cocaine place preference
Carbo-Gas et al., 2017	Substance Use Disorders (SUD)	Cocaine induces differential PNN changes in cerebellar Golgi neurons and projection neurons
Donegan and Lodge, 2017	Major Depression Disorder (MDD)	PNN degradation in the ventral hippocampus of rats impairs the long-term efficacy of low dose ketamine
Riga et al., 2017	Major Depression Disorder (MDD)	Increased CSPG proteins and PNNs and decreased inhibitory postsynaptic currents occur in the hippocampus of rats exposed to chronic social defeat stress
Steullet et al., 2017	Schizophrenia (SZ)	PNN and PVB neuron reduction occurs in the TRN of SZ subjects
Foscarin et al., 2017	Alzheimer’s Disease (AD)	CS-4 levels are increased in aged in rats, associated with cognitive deficits and restricted axonal growth
Yang et al., 2017	Alzheimer’s Disease (AD)	Antibody targeting of CS-4 temporarily improves novel object recognition memory in the P301S mouse model of AD
Egervari et al., 2017	Substance Use Disorders (SUD)	Altered gene expression of several ECM molecules in the striatum of people with heroin abuse, including ACAN, CSPG4, MMP28, and ADAMTS3.
Vazquez-Sanroman et al., 2017	Substance Use Disorders (SUD)	Nicotine self-administration alters PNNs in the rat ventral tegmental area and orbitofrontal cortex
Stefaniuk et al., 2017	Substance Use Disorders (SUD)	MMP-9 expression in the central amygdala regulates alcohol seeking behavior in rodents
Bach et al., 2018	Post-Traumatic Stress Disorder (PTSD)	Doxycycline, an MMP inhibitor, reduces arousal and fear memory in humans
Blacktop and Sorg, 2018	Substance Use Disorders (SUD)	PNNs in the lateral hypothalamic area are necessary for cue induced reinstatement of cocaine seeking behavior in rodents
Alaiyed et al., 2019	Major Depression Disorder (MDD)	Venlafaxine, a serotonin/norepinephrine reuptake inhibitor, increases MMP-9 expression and decreases PNN composition in rodents
Alcaide et al., 2019	Major Depression Disorder (MDD)	PNN density is unaltered in the dorsolateral prefrontal cortex of subjects with MDD
Alaiyed et al., 2020	Major Depression Disorder (MDD)	Increased MMP-9 expression and reduced PNN composition after venlafaxine treatment are associated with increased hippocampal gamma activity; venlafaxine efficacy may occur through MMP-9 degradation of PNNs
Crapser et al., 2020	Alzheimer’s Disease (AD)	Activated microglia engulf PNNs in AD; microglial depletion prevents PNN engulfment
Koskinen et al., 2020	Major Depression Disorder (MDD)	Increases in hippocampal PNN intensity develop 4–8 weeks after social defeat stress, and coincide with spatial memory deficits
Nguyen et al., 2020	Alzheimer’s Disease (AD)	IL-33 promotes ECM degradation for memory processing, and IL-33 is reduced with age in mice, associated with memory deficits. Rescue of IL-33 signaling restores memory performance.
Pantazopoulos et al., 2020b	Schizophrenia (SZ)	In SZ, broad irregularities of ECM molecule gene expression, including CSPG core proteins, chondroitin sulfate synthesis pathways, endogenous proteases, is associated with cognitive deficits
Yu et al., 2020	Major Depression Disorder (MDD)	PNN density is reduced in the prelimbic cortex in rats exposed to chronic mild unpredictable stress that display depression-like behavior
Seney et al., 2020	Substance Use Disorders (SUD)	Altered gene expression of several ECM molecules in the DLPFC and VTA of human subjects with opioid abuse.

### Schizophrenia

Synaptic deficits and sleep dysfunction are both widely reported in SZ. Decreased dendritic spines on pyramidal neurons are an established feature of SZ and have been reported in several brain regions, including the dorsolateral prefrontal cortex, visual cortex, and striatum (Roberts et al., 1996; Garey et al., 1998; Glantz and Lewis, 2000; Konopaske et al., 2014; Parker and Sweet, 2017; McKinney et al., 2019). A recent meta-analysis of human postmortem studies provides additional support for reduced synapses in cortical regions in SZ (Berdenis van Berlekom et al., 2020). Deficits in molecular synaptic markers, particularly for NMDA receptors, have also been reported in several brain regions in SZ (Woo et al., 2004; Kristiansen et al., 2010; Schmitt et al., 2010; Vrajova et al., 2010). Sleep and circadian rhythm disturbances may contribute to synaptic deficits in SZ. Sleep deprivation has been shown to result in decreased long-term plasticity (LTP) in CA1 hippocampal neurons, associated with reduced NMDA receptor activity and a reduced surface expression of the NR1 and NR2A subunits of the NMDA receptor in sleep deprived rats, indicating that sleep deprivation prevents translocation of NMDA receptors to the cell surface (McDermott et al., 2006).

Recent studies point to sleep and circadian rhythm disturbances as key aspects of schizophrenia (SZ) (Ferrarelli et al., 2007; Manoach et al., 2014, 2016; Ferrarelli, 2015, 2020; Ferrarelli and Tononi, 2017; D’Agostino et al., 2018; Baran et al., 2019; Manoach and Stickgold, 2019; Merikanto et al., 2019; Seney et al., 2019; Delorme T.C. et al., 2020; Gerstenberg et al., 2020; Markovic et al., 2020; Meyer et al., 2020). Furthermore, altered diurnal molecular expression rhythms have been recently reported in the prefrontal cortex of subjects with SZ using RNAseq profiling of postmortem brain samples, suggesting broad molecular circadian rhythm disturbances in this disease (Seney et al., 2019). Disrupted sleep and circadian rhythms may impair synaptic downscaling of unnecessary spines during sleep (Tononi and Cirelli, 2006, 2014), and strengthening of selective spines for enhancement of specific memories (Rasch and Born, 2013; Yang et al., 2014; Dudai et al., 2015). Despite the clinical and genetic heterogeneity of SZ (Fanous et al., 2012; Cross-Disorder Group of the Psychiatric Genomics Consortium et al., 2013), sleep dysfunction, specifically decreased sleep spindles and memory consolidation deficits, is emerging as a consistent characteristic of the disease (Ferrarelli et al., 2007; Manoach et al., 2014, 2016; Ferrarelli, 2015, 2020; Ferrarelli and Tononi, 2017; D’Agostino et al., 2018; Baran et al., 2019; Manoach and Stickgold, 2019; Merikanto et al., 2019; Gerstenberg et al., 2020; Markovic et al., 2020). Decreases in sleep spindles are found together with memory consolidation deficits in patients with SZ (Ferrarelli et al., 2007; Manoach et al., 2010, 2014). Sleep spindle deficits have been reported in several independent studies (Ferrarelli et al., 2007; Manoach et al., 2014, 2016; Ferrarelli, 2015, 2020; Ferrarelli and Tononi, 2017; D’Agostino et al., 2018; Baran et al., 2019; Manoach and Stickgold, 2019; Merikanto et al., 2019; Gerstenberg et al., 2020; Markovic et al., 2020) including in unmedicated patients with SZ, and in first-degree relatives, suggesting that this represents a core genetic component of the disease rather than medication effects or a degenerative consequence of disease progression. Sleep spindle deficits are highly associated with psychosis, and disease prognosis (Manoach et al., 2016; Gerstenberg et al., 2020; Markovic et al., 2020).

Circadian rhythms in PNN composition may impact regulation of sleep spindles and memory consolidation in SZ in several ways. Our group and others have reported PNN deficits in several brain regions in SZ (Pantazopoulos et al., 2010b, 2014, 2015; Mauney et al., 2013; Enwright et al., 2016; Steullet et al., 2017). Furthermore, we recently reported broad alterations of ECM molecule gene expression in subjects with SZ associated with cognitive deficits, impacting CSPG core proteins, chondroitin sulfate synthesis pathways, and endogenous proteases across several cortical and subcortical brain regions (Pantazopoulos et al., 2020b). Recent evidence for altered diurnal molecular expression rhythms of subjects with SZ (Seney et al., 2019) suggests broad molecular circadian rhythm disturbances, which may impact several of these memory consolidation processes in which ECM molecules are involved. Broad ECM abnormalities, and potentially disrupted expression rhythms in ECM molecules in SZ, may impact PNNs and other CSPGs that regulate synaptic function and NMDA receptor trafficking, and may thus contribute to memory consolidation deficits through disruption of synaptic regulation proposed to occur during sleep (Tononi and Cirelli, 2006; Rasch and Born, 2013; Tononi and Cirelli, 2014; Puentes-Mestril and Aton, 2017; Havekes and Aton, 2020). In turn, these memory consolidation processes may contribute to synaptic deficits in SZ (Penzes et al., 2011; Glausier and Lewis, 2013; Shelton et al., 2015; MacDonald et al., 2017).

Circadian rhythms in PNN composition may also impact sleep spindles in SZ. PNN PNNs regulate firing rates of neurons expressing parvalbumin (PVB), including those in the thalamic reticular nucleus (TRN) that generate sleep spindles (Csillik et al., 2005; Katsuki et al., 2017; Latchoumane et al., 2017; Fernandez et al., 2018; Thankachan et al., 2019). Decreases of PNNs and PVB neurons were detected in the TRN of subjects with schizophrenia (Steullet et al., 2017). Potentially disrupted PNN rhythms in this region may impair the ability of the TRN to generate sleep spindles, impacting memory consolidation processes.

### Major Depression and Bipolar Disorder

Decreases of dendritic spines and synaptic markers have been reported in several brain regions in BD and MDD, including the prefrontal cortex and hippocampus (Feyissa et al., 2009; Kang et al., 2012; Duric et al., 2013; Licznerski and Duman, 2013; Konopaske et al., 2014, 2015), and increased synaptic markers were reported in the lateral amygdala of subjects with MD (Karolewicz et al., 2009). Abnormalities in sleep and circadian rhythms have been consistently reported in these disorders (Leibenluft et al., 1996; Colombo et al., 1999; Jackson et al., 2003; Harvey et al., 2005; Armitage, 2007; Benedetti et al., 2007; McCarthy et al., 2012, 2013, 2016, 2019; Bunney and Bunney, 2013; Burton et al., 2013; Li et al., 2013; McClung, 2013; Palagini et al., 2013; Medina et al., 2014; Bunney et al., 2015; Pagani et al., 2016; Pantazopoulos et al., 2017, 2018; Nudell et al., 2019). These findings include diurnal rhythms in symptom severity, with anxiety and depression peaking in the morning (Murray et al., 2002; Murray, 2007, 2008; Wirz-Justice, 2008). Disrupted circadian rhythms in locomotor activity associated with several genetic factors have been reported in subjects with BD (Pagani et al., 2016). Disrupted gene expression diurnal rhythms have been reported in the brain of subjects with MDD (Bunney and Bunney, 2013; Li et al., 2013; Bunney et al., 2015). Altered diurnal rhythms of the anxiolytic neurotransmitter somatostatin were also reported in the amygdala of subjects with BD (Pantazopoulos et al., 2016), with decreased levels of somatostatin occurring in the morning, coinciding with reported increased severity of anxiety and depression (Murray et al., 2002; Murray, 2007, 2008; Wirz-Justice, 2008).

Animal models targeting the circadian system suggest that sleep and circadian rhythms are causative factors of mood disturbances, including mania, depression, and anxiety (Roybal et al., 2007; Dulcis et al., 2013; Landgraf et al., 2016). Conversely, chronic stress in mice also results in sleep and circadian rhythm disturbances reflective of findings in humans (Meerlo et al., 1996, 1997; Keeney et al., 2001; Henderson et al., 2017; Wells et al., 2017; Ota et al., 2018; Moravcova et al., 2020), without altering the molecular clock in the suprachiasmatic nucleus (Ota et al., 2018; Ota et al., 2020).

Pharmacological treatments, and genetic factors associated with treatment efficacy, point to a critical role of circadian rhythms in mood disorders. Gene association studies, including GWAS, identified gene variants for clock and clock controlled molecules associated with BD and lithium responsiveness (Nievergelt et al., 2006; Mansour et al., 2009; Sjoholm et al., 2010; Soria et al., 2010; McCarthy et al., 2012; McCarthy and Welsh, 2012; Pandey et al., 2012; Rybakowski, 2014; Bollettini et al., 2017; Suzuki et al., 2017). Furthermore, lithium and valproic acid, among the most common and effective treatments for mood disorders, alter core clock molecule expression and modulate the period length and amplitude of molecular circadian rhythms (Klemfuss, 1992; Abe et al., 2000; Yin et al., 2006; Johansson et al., 2011; Li et al., 2012; Noguchi et al., 2016). CACNA1C, one of the genes strongly associated with BD, is involved in regulating circadian rhythms through L-type calcium channels (Ferreira et al., 2008; Sklar et al., 2008; Liu et al., 2011; Kumar et al., 2013; Fiorentino et al., 2014; Schmutz et al., 2014; Nudell et al., 2019). Growing evidence also indicates that CACNA1C is involved in efficacy of pharmacological therapies for BD. For example, the ability of lithium to amplify circadian rhythms is dependent on L-type calcium channels including CACNA1C, and the effectiveness of lithium on restoring circadian amplitude in skin fibroblasts from patients with BD is dependent on genotype, including CACNA1C genotype (McCarthy et al., 2016; Nudell et al., 2019). Light therapy also represents a promising strategy for the treatment of mood disorders (Sit et al., 2007; Henriksen et al., 2014, 2016, 2020; Kaladchibachi and Fernandez, 2018; Sit et al., 2018). Specifically, the efficacy of bright light therapy for depression (Sit et al., 2007, 2018; Kaladchibachi and Fernandez, 2018) and blue light blocking treatment for mania (Henriksen et al., 2014, 2016, 2020) suggest that patient specific approaches targeting the circadian system are promising therapeutic strategies.

Several lines of evidence suggest that PNNs are critically involved in disease symptoms and treatment efficacy in BD and MDD. Chronic social defeat stress in rats, commonly used to model depression, results in increased CSPG proteins and PNN numbers in the hippocampus, together with reduced frequency of inhibitory postsynaptic currents (Riga et al., 2017). These PNN changes emerge 4–8 weeks after social defeat stress, and coincide with the emergence of later stage spatial memory deficits (Koskinen et al., 2020). Chronic social defeat also results in changes in sleep, circadian rhythm, and clock gene measures in mice that persist afterward (Wells et al., 2017), suggesting that PNN alterations may be influenced by circadian rhythm disruption. The antidepressants venlafaxine, a serotonin/norepinephrine reuptake inhibitor, has been shown to increase MMP-9 expression in the rodent brain and reduce PNN composition (Alaiyed et al., 2019), and subjects with MDD with antidepressant treatment have increased MMP-9 expression in the dorsolateral prefrontal cortex compared to control subjects (Alaiyed et al., 2020). These changes in MMP-9 and PNNs following venlafaxine treatment were also associated with increased hippocampal gamma activity, suggesting that antidepressant efficacy of venlafaxine may occur in part through MMP-9 reduction of PNN composition (Alaiyed et al., 2020). Chronic treatment with the antidepressant fluoxetine has also been shown to reduce PNNs in mice, along with alterations in synaptic markers and dendritic spines (Guirado et al., 2014), providing further support that antidepressant efficacy may in part be due to promotion of synaptic plasticity through PNN degradation. Furthermore, chemical degradation of PNNs in the ventral hippocampus impairs the long-term antidepressant effect of low dose ketamine in rats (Donegan and Lodge, 2017), suggesting that PNN composition deficits, and/or alterations in PNN composition rhythms in these disorders, may impact treatment efficacy.

Perineuronal net deficits have been reported in the dorsolateral prefrontal cortex, amygdala and TRN in BD (Pantazopoulos et al., 2015; Steullet et al., 2017; Alcaide et al., 2019). Despite the evidence from rodent studies strongly suggesting that PNNs are altered in depression, and that antidepressant efficacy in part is associated with PNN degradation, current evidence for PNN alterations in MDD is limited. Currently, one human postmortem study reported lack of changes in PNN density in the dorsolateral prefrontal cortex of subjects with MDD (Alcaide et al., 2019). Disruption of PNNs in these disorders may alter rhythms of synaptic plasticity and in turn contribute to shared synaptic deficits (Penzes et al., 2011; Glausier and Lewis, 2013; Shelton et al., 2015; MacDonald et al., 2017). Such deficits may arise from disrupted memory consolidation processes allowing for decreased synaptic formation and/or increased synaptic pruning in brain regions involved in emotional memory processing.

### Post-traumatic Stress Disorder

Enhanced fear memories are a core feature of post-traumatic disorder (PTSD) (Parsons and Ressler, 2013). Similarly, sleep disruption and nightmares are common symptoms in people with PTSD, and are associated with heightened strength of fear memories (Ross et al., 1989; Raskind et al., 2007; Thompson et al., 2008; Harb et al., 2012; Brownlow et al., 2015). Studies suggest that these sleep disturbances contribute to the persistent heightened fear memories in this disorder. For example, frequent nightmares and SD in patients suffering from PTSD is associated with impaired extinction of fear memories (Mellman et al., 2002). Sleep deprivation, particularly decreased REM sleep, impairs fear extinction learning in both rodents and humans (Silvestri, 2005; Fu et al., 2007). In contrast, studies suggest that sleep deprivation early on following a traumatic experience may help to alleviate the strength of fear memories that contribute to PTSD (Wagner et al., 2006; Kuriyama et al., 2010; Cohen et al., 2012). Sleep after learning enhances emotional memories in humans for up to 4 years (Wagner et al., 2006), and sleep deprivation reduces emotional memories in human subjects (Kuriyama et al., 2010). Rodent studies provide additional support and point to potential mechanisms for this process. For example, sleep deprivation reduces fear memory and alters glucocorticoid signaling and dendritic spines in the hippocampus (Cohen et al., 2012).

Chronic stress can serve as a predisposing factor for PTSD (Davidson et al., 1991; Breslau et al., 1999; Chetty et al., 2014; Meyer et al., 2014; Brockie et al., 2015; Yousufzai et al., 2018). Rodent models of chronic stress suggest that predisposition may be due in part to changes in dendritic spines. Chronic stress in rodents decreases dendritic spines in the hippocampus in comparison to increased spines in the amygdala, which may underlie generalization of fear response (Vyas et al., 2002). Similarly, chronic stress was shown to increase ghrelin-growth hormone signaling (Meyer et al., 2014), which can increase amygdala dendritic spines and predispose these neurons to encode fear memories (Gisabella et al., 2016). Studies of humans with chronic stress showing provide further support for the neurocircuitry effects of chronic stress suggested by rodent studies. Subjects with chronic stress display decreased hippocampal synaptic markers and hippocampal volume (Lange and Irle, 2004; Videbech and Ravnkilde, 2004; Smith, 2005; Soetanto et al., 2010). In comparison, increased amygdala volume and activity (Bremner et al., 2000; Frodl et al., 2002; Drevets, 2003; Lange and Irle, 2004; Tottenham et al., 2010) are reported in chronic stress subjects, possibly reflecting increased dendritic spines. Several studies suggest that SD following emotional learning may be a promising preventative measure for PTSD (Kuriyama et al., 2010; Cohen et al., 2012, 2017).

Perineuronal nets are strongly involved in fear memory processing (Gogolla et al., 2009; Banerjee et al., 2017). Regulation of PNN composition represent a therapeutic strategy for weaking fear memories that are enhanced in people suffering from PTSD. Disruption of molecular processes involved in PNN rhythms may represent one of the potential mechanisms through which sleep deprivation may impact memory consolidation (Kuriyama et al., 2010; Cohen et al., 2012, 2017) and may possible therapeutic approach for alleviating the strength of fear memories contributing to PTSD. Intracerebroventricular administration of the MMP inhibitor FN-439 has been shown to disrupt fear memory reconsolidation in rodents (Brown et al., 2009), and MMP-9 knockout mice display deficits in LTP and fear memory (Nagy et al., 2006). Furthermore, the MMP inhibitor doxycycline reduces fear memory and arousal in humans (Bach et al., 2018). These findings suggest that inhibition of endogenous proteases that may block regulation of PNN composition and other ECM components during sleep may impair normally occurring consolidation processes during sleep that strengthen emotional memories following a traumatic experience. Subsequently, the development of nightmares and sleep disturbances may impact the ability to extinguish fear memories later on. Further regarding the timing of administration of endogenous protease inhibitors, both in terms of distance from the traumatic event and as well as timing of the sleep-wake cycle will provide insight into the potential ECM protease inhibitors as therapeutic strategies for PTSD.

### Substance Abuse Disorders

Sleep disturbances are associated in a bi-directional manner with the cycle of impulsivity and cognitive deficits in substance use disorders (Koob and Colrain, 2020). Drugs of abuse cause direct phase-shifting of circadian rhythm and alter sleep architecture when used shortly before sleep (Lin et al., 2000; Wang and Teichtahl, 2007; Colrain et al., 2014; Narishige et al., 2014). A hallmark of SUD is abnormalities and inconsistency in chronotype, and adolescent sleep deprivation is a risk factor for developing a substance use disorder later in life (Pasch et al., 2012). Preclinical studies investigating the impact of sleep and circadian rhythm abnormalities on behavioral responses to drugs of abuse indicate that sleep restriction increases reward seeking. REM sleep deprivation enhances ethanol self-administration (Aalto and Kiianmaa, 1986). Conversely, 6 h of sleep deprivation following re-exposure to morphine reward impairs reward memory reconsolidation (Shi et al., 2011), indicating sleep plays a central role in the regulation of reward memory consolidation.

Extracellular matrix molecules alterations have been extensively described as a key feature of reward memory processing (Xue et al., 2014; Slaker et al., 2015; Blacktop and Sorg, 2018). Altered expression of ECM molecules has been reported in several human postmortem brain studies, including in the striatum of people with heroin abuse (Egervari et al., 2017)and in the dorsolateral prefrontal cortex and ventral tegmental area of subjects with opioid abuse (Seney et al., 2020). PNNs are well represented in neural circuits involved in emotion processing and critically involved in the regulation of fear and reward memories (Gogolla et al., 2009; Slaker et al., 2015; Banerjee et al., 2017; Lasek et al., 2018). Alteration in PNNs has been described as a key feature in addiction (Xue et al., 2014; Slaker et al., 2015; Vazquez-Sanroman et al., 2017; Blacktop and Sorg, 2018). Ablation of PNNs in the corticostriatal circuit significantly weakens drug reward memories, indicating that broad PNN and ECM density increases are a molecular signature of addiction (Brown et al., 2007). While there are increases in PNN densities after prolonged drug use, initial acquisition of drug memories appears to rely on the activity of MMPs to degrade PNN components and allow for formation of new synapses (Wright et al., 2003; Mizoguchi et al., 2007; Smith et al., 2011, 2014; Kovatsi et al., 2013; Natarajan et al., 2013; Stefaniuk et al., 2017). Modulation of PNNs is necessary for reward memory acquisition, and prolonged re-activation of drug-associated engrams reconsolidates PNN components, stronger than at baseline, to stabilize and potentiate these synapses (Van den Oever et al., 2010; Chen et al., 2015; Slaker et al., 2015, 2018; Blacktop et al., 2017; Carbo-Gas et al., 2017). For example, broad spectrum MMP inhibition disrupts both acquisition and consolidation of cocaine reward memory in rats (Brown et al., 2007). Furthermore, a genetic polymorphism for MMP-9 is associated with alcoholism (Samochowiec et al., 2010). Circadian rhythms in PNN composition may have a significant impact in reward memory processing. PNN composition decreases during the inactive phase may contribute to enhanced reward sensitivity in the evening reported in human subjects (Murray et al., 2009; Pantazopoulos et al., 2020a). PNN digestion in the ventral hippocampus by chondroitinase ABC enhances dopamine VTA activity and amphetamine response (Shah and Lodge, 2013), providing further support that PNN composition decreases during the inactive phase may contribute to enhanced reward sensitivity in the evening in humans (Murray et al., 2009). Disruption of PNN composition rhythms in turn may impair the memory consolidation processes that normally occur during sleep (Tononi and Cirelli, 2006; Rasch and Born, 2013; Tononi and Cirelli, 2014; Dudai et al., 2015), and thus may represent a promising therapeutic target for drug addiction.

## Aging and Alzheimer’s Disease

Synaptic deficits are a hallmark of aging and Alzheimer’s Disease (DeKosky and Scheff, 1990; Terry et al., 1991; Scheff and Price, 1993; Kristiansen et al., 2010; Schmitt et al., 2010). Disrupted sleep and circadian rhythm have also been consistently described with age (Bliwise, 1993; Van Cauter et al., 2000; Huang et al., 2002; Harper et al., 2005; Chen et al., 2016) and are key features of Alzheimer’s Disease (Witting et al., 1990; Volicer et al., 2001; Harper et al., 2005; Ju et al., 2013; Musiek et al., 2018). For example, circadian rhythm amplitude decreases with age as people shift to an early bird chronotype (Huang et al., 2002). Aging is also characterized by increased insomnia and fragmented sleep (Bliwise, 1993; Van Cauter et al., 2000). Alterations are also observed in expression of genes and proteins in the brain involved in the regulation of circadian rhythms (Hofman et al., 1996; Wang et al., 2015; Chen et al., 2016). Diurnal expression rhythms of clock genes in the brain are impaired with age in humans (Chen et al., 2016). SCN neurons expressing the neurotransmitter vasoactive intestinal peptide (VIP), which is important to maintaining strength of circadian rhythmicity (Aton et al., 2005; Pantazopoulos et al., 2010a; Jones et al., 2018; Mazuski et al., 2018, 2020), are decreased with age in the human SCN (Hofman et al., 1996), and are correlated with the strength of locomotor circadian rhythms recorded prior to death (Wang et al., 2015). Disruptions in molecular circadian rhythms with age may also contribute to disruption of ECM processes involved in synaptic regulation during sleep, thus impairing memory consolidation processes. Impaired sleep spindles and memory consolidation with age support this hypothesis (Guazzelli et al., 1986; DeKosky and Scheff, 1990; Terry et al., 1991; Scheff and Price, 1993; Crowley et al., 2002; Knoblauch et al., 2005).

Decreases of PNNs have been reported in AD patients and mouse models (Baig et al., 2005; Crapser et al., 2020). A recent study combining analysis of human postmortem brain samples and an animal model of AD indicates that activated microglia engulf PNNs in AD, and microglial depletion prevents this engulfment (Crapser et al., 2020). In contrast, other studies suggest that PNNs are protective factors for neurons in AD (Morawski et al., 2004, 2010). Evidence regarding changes in levels of sulfated CS point to more consistent findings in AD and aging. CS-4 sulfation inhibits axonal growth (Smith-Thomas et al., 1995; Wang et al., 2008), whereas CS-6 is much more permissive for axonal growth (Lin et al., 2011; Miyata et al., 2012), in part through promoting degradation of the major PNN component aggrecan by ADAMTS (Miyata and Kitagawa, 2016). The ratio of CS-4/CS-6 increases with age, suggesting that this increase in CS-4 contributes to restricted plasticity in aging (Foscarin et al., 2017). Preclinical studies in mice suggest that targeting CS-4 may improve cognition in Alzheimer’s disease (Yang et al., 2017). Recent work demonstrates that neurons signal to microglia through IL-33 to stimulate engulfment of ECM, which promotes synapse formation (Nguyen et al., 2020). In aged animals this process is impaired due to less IL-33, which results in reduced synapses and impaired spatial memory resolution (Nguyen et al., 2020). Restoration of IL-33 signaling restores younger numbers of dendritic spines and spatial memory performance (Nguyen et al., 2020). This evidence is in-line with studies that suggest CS-4 accumulation with age restricts synaptic plasticity and contributes to memory deficits (Foscarin et al., 2017; Yang et al., 2017). Disruption of circadian rhythms with age may impair circadian regulation of PNNs, contributing to memory consolidation and synaptic deficits reported in aging and Alzheimer’s Disease (Guazzelli et al., 1986; DeKosky and Scheff, 1990; Terry et al., 1991; Scheff and Price, 1993; Crowley et al., 2002; Knoblauch et al., 2005; Kristiansen et al., 2010; Schmitt et al., 2010).

## Conclusion

In summary, sleep and synaptic dysfunction is present across psychiatric disorders. Extracellular matrix molecules are involved in synaptic regulation and their expression is disrupted in these disorders. Recent studies point to the critical involvement of ECM molecules including PNNs in memory consolidation, thus ECM molecules represent promising therapeutic targets for memory consolidation dysfunction in psychiatric disorders.

## Author Contributions

All authors contributed to literature review and manuscript writing. BG and HP wrote the first draft and edited the manuscript.

## Conflict of Interest

The authors declare that the research was conducted in the absence of any commercial or financial relationships that could be construed as a potential conflict of interest.
